# Physical fitness of primary school children differs depending on their timing of school enrollment

**DOI:** 10.1038/s41598-023-35727-y

**Published:** 2023-05-31

**Authors:** Paula Teich, Thea Fühner, Urs Granacher, Reinhold Kliegl

**Affiliations:** 1 Division of Training and Movement Science, Faculty of Human Sciences, University of Potsdam, Am Neuen Palais 10, Building 12, 14469 Potsdam, Germany; 2 Exercise and Human Movement Science, Department of Sport and Sport Science, University of Freiburg, Freiburg, Germany

**Keywords:** Paediatrics, Physiology, Health care

## Abstract

Previous research has shown that children who were enrolled to school according to the legal key date (i.e., keyage children, between eight and nine years in third grade) exhibited a linear physical fitness development in the ninth year of life. In contrast, children who were enrolled with a delay (i.e., older-than-keyage children [OTK], between nine and ten years in third grade) exhibited a lower physical fitness compared to what would be expected for their age. In these studies, cross-sectional age differences within third grade and timing of school enrollment were confounded. The present study investigated the longitudinal development of keyage and OTK children from third to fifth grade. This design also afforded a comparison of the two groups *at the same average chronological age*, that is a dissociation of the effects of timing of school enrollment and age. We tested six physical fitness components: cardiorespiratory endurance, coordination, speed, power of lower and upper limbs, and static balance. 1502 children (i.e., 1206 keyage and 296 OTK children) from 35 schools were tested in third, fourth, and fifth grade. Except for cardiorespiratory endurance, both groups developed from third to fourth and from fourth to fifth grade and keyage children outperformed OTK children at the average ages of 9.5 or 10.5 years. For cardiorespiratory endurance, there was no significant gain from fourth to fifth grade and keyage and OTK children did not differ significantly at 10.5 years of age. One reason for a delayed school enrollment could be that a child is (or is perceived as) biologically younger than their chronological age at the school entry examination, implying a negative correlation between chronological and biological age for OTK children. Indeed, a simple reflection of chronological age brought the developmental rate of the chronologically youngest OTK children in line with the developmental rate observed for keyage children, but did not eliminate all differences. The mapping of chronological and biological age of OTK children and other possible reasons for lower physical fitness of OTK children remain a task for future research.

## Introduction

Children’s cardiorespiratory endurance and muscular fitness are related to various health outcomes in childhood and later in life. Low fitness levels are associated with cardiometabolic risk factors^1–3^, such as percent body fat^2,4^, obesity^5,6^, or insulin resistance^3^. High levels of muscular fitness (maximal strength and power, local muscular endurance) are related to higher bone mineral density^7,8^. The role of children’s physical fitness as a health indicator extends to psychological and cognitive outcomes, with studies reporting a positive association with health-related quality of life^9–11^, executive function^12–14^, relational memory^15^, and academic performance^16–19^. As physical fitness in youth moderately predicts physical fitness in adulthood^20,21^, identifying children with low fitness levels and assigning them to early fitness-promoting interventions can set an important foundation for long-term health.

A strong predictor for physical fitness in childhood is children’s age^22^, and there is evidence that physical fitness may vary depending on the timing of school enrollment^23,24^. The same school grade spans a wide age range of children due to factors such as timing of school enrollment or the skipping or repetition of a grade. Compulsory school attendance in Germany applies to children who have turned six before a key date. In the Federal State of Brandenburg, all children who reach the age of six by September 30 (i.e., key date) are required to attend primary school on August 1 of the same year^25^. However, the school enrollment of a child can be delayed if pedagogical experts, parents or health care professionals decide that a child would benefit from one more year in kindergarten, for example due to developmental delays. Conversely, a child who is an early developer can be enrolled in school even earlier than foreseen by law. The same school grade can thus include children with timely school enrollment (i.e., keyage children), and children who are younger or older than keyage children (i.e., older-than-keyage children, OTK).

Keyage third-graders exhibit a remarkably linear age-related development in the five physical fitness components cardiorespiratory endurance, coordination, speed, lower, and upper limbs muscle power (i.e., powerLOW and powerUP)^22^. In a sample of 108,295 keyage third-graders from nine cohorts, the age-related gains were different for the five components, but statistically parallel for boys and girls. However, OTK third-graders (i.e., between 9.0 and 10.0 years old) exhibited significantly poorer physical fitness compared to what would be expected for their age^23^. Using the growth rates of keyage boys and girls, Fühner et al.^23^ predicted the performance of 26,540 OTK third-graders with a linear mixed effects model (i.e., LMM; taking into account  school- and cohort-related random effects) and compared the observed physical fitness with test scores extrapolated from the LMM. In this cross-sectional study, OTK children performed below their predicted performance in all fitness tests (i.e., 6-min run test, star-run coordination test, 20-m sprint, standing long jump, and ball-push test). Similar findings were reported in a study using the German Motor Test^26^, where ten-year-old third-graders from Berlin, Germany, exhibited poorer performance in several fitness tests (i.e., including the 6-min-run test, 20-m sprint, and standing long jump test) compared to their younger peers^24^.

What are the reasons for the discrepancy between the predicted and observed physical fitness of OTK children? One hypothesis is that they are developmentally delayed and were held back for a good reason. Developmental delays may be the result of the non-linearity of maturational processes^27^, meaning that children are biologically younger than indicated by their chronological age. They can also be related to socioeconomic disadvantage^28^. Although there is little research on the physical development of OTK, studies regarding cognitive development of OTK children^29–32^ seem to be in line with the hypothesis of developmental delays. For instance, previous studies have reported a lower academic performance for OTK children, compared to keyage children. In a sample of 15,707 German third- and fourth-graders with a mean age of 9.9 years, ordinary least squares regressions (OLS) showed lower math skills for students with delayed school enrollment, compared to their younger peers^29^. Similarly, Puhani and Weber^31^ examined the reading skills of 6591 German fourth-graders. OLS regressions yielded negative associations for age at school entry with reading skills. In these studies, the effect sizes of the timing of school enrollment decreased when covariates associated with the socioeconomic status (e.g., parental education, migration status, number of books at home) were added to the models, pointing to factors possibly associated with developmental delays of OTK children. In fact, there is evidence that lower family socioeconomic status and higher regional socioeconomic deprivation increases the odds for global developmental delays (assessed via language and cognitive abilities) at school entry examination^28^.

An alternative hypothesis for the lower physical fitness of OTK children relative to their predicted performance, is that physiological changes due to biological maturation (e.g., rise of circulating anabolic hormones and changes in body composition^33^) or due to psychological factors (e.g., motivation) cause a somewhat abrupt stagnation of physical fitness development in the tenth year of life. Thus, if fitness development slowed down after the age of 9.0 years, the extrapolated test scores based on data from children aged 8.0–9.0 years in the analyses conducted by Fühner et al.^23^ would overestimate the performance of children who are one year older. The observed performance of OTK children aged 9.0–10.0 years might actually indicate ‘normal’ physical fitness development during this short time span, instead of the reported physical fitness deficit.

These hypotheses cannot be tested using within-grade cross-sectional data such as those reported in previous research^23,24^. From within-grade cross-sectional data, we cannot determine whether the observed physical fitness of OTK children indeed indicates a delayed physical fitness development, or is due to physiological or psychological changes occurring in the tenth year of life. In other words, there is a confound of ‘timing of school enrollment’ and ‘age’. In the present study, we therefore used a longitudinal design to (1) compare the physical fitness of OTK and keyage children *at the same chronological age* (i.e., implying a comparison across different school grades), and to (2) examine the longitudinal development of keyage and OTK children across three school grades. Specifically, we compared the physical fitness of OTK third-graders to the physical fitness of age-matched keyage fourth-graders, and the physical fitness of OTK fourth-graders to the physical fitness of age-matched keyage fifth-graders. The simple question was: Do OTK children exhibit a lower physical fitness compared to age-matched keyage children? The longitudinal nature of the study further allowed us to examine whether potential fitness deficits of OTK children increased or decreased over time relative to the physical fitness of keyage children. Based on previous research on OTK children’s physical fitness^23,24^ and cognitive development^29–32^, we hypothesized that OTK children would exhibit poorer physical fitness than age-matched keyage children.

## Methods

### Experimental approach

The EMOTIKON research project (uni-potsdam.de/en/emotikon/) annually tests the physical fitness of children in the Federal State of Brandenburg, Germany. EMOTIKON was mandated and approved by the Ministry of Education, Youth and Sport of Brandenburg. According to the Brandenburg School Law, participation is obligatory for all third-graders in public primary schools^25^. In addition to the cross-sectional assessment of third-graders, a longitudinal assessment of children’s physical fitness is being carried out in Potsdam-Mittelmark, one of 18 districts of the Federal State of Brandenburg. Children in Potsdam-Mittelmark who were tested as third-graders in 2020 were tested again one year later in 2021 as fourth-graders and another year later in 2022 as fifth-graders.

The physical fitness tests were conducted between August and December of 2020, 2021 and 2022. Prior to testing, schools and parents received written information about the EMOTIKON study, including risks and benefits, instructions on test administration and information on data processing and data protection. Research was conducted in accordance with the latest Declaration of Helsinki^34^ and the Brandenburg School Law^25^. The authors received the data completely anonymized from the Ministry of Education, Youth and Sport of the Federal State of Brandenburg, Germany. None of the authors had access to personally identifiable information of the children.

### Participants

Overall, 1,956 children living in the district Potsdam-Mittelmark participated in at least one assessment in 2020, 2021 or 2022. In 2020, 1,565 third-graders from 35 schools participated in the study. In 2021, 1436 fourth-graders from 32 schools participated in the study. In 2022, 1422 fifth-graders from 35 schools were tested. Allocation to the groups of keyage or OTK children was determined using the children’s ages relative to the key date of school enrollment in the Federal State of Brandenburg (i.e., September 30). As grade repetitions in the first years of primary school are very rare (around 1% in third grade), and as percentage of delayed school enrollments is much higher (around 16% in school year 2018/19)^35^, it is likely that most OTK children in our sample were enrolled to school with a delay.

For each physical fitness test, except for the one-legged-stance test, we excluded test scores outside of a ± 3 *SD* range that was determined separately for boys and girls. For the one-legged-stance test, scores outside of a ± 3 *SD* range are valid and do not indicate measurement error. This left us with 25,210 test scores from 1955 children. We only kept data from children who were tested in at least two assessment years. This left us with a final sample of 1502 children from 35 different schools (i.e., 1206 keyage and 296 OTK children; 22,593 test scores). With a few exceptions, the pattern of results did not change when we limited analyses to 965 children who were tested in all three assessment years. The exceptions were five estimates that were not significant with the smaller sample; these effects were not in the focus of this paper (details on this LMM are reported in script *LS_lmm.qmd* in the OSF repository).

Twenty-two of the 1502 children of the final sample were younger than keyage children (mean age in third grade 7.84 years), either due to early school enrollment or skipping a grade. Due to their small number, we included them in our group of keyage children; however, the pattern of results did not change when we excluded them from analyses (details on this LMM are reported in script *LS_lmm.qmd* in the OSF repository). Table 1 shows the number of keyage and OTK children, schools and observations (i.e., test scores) in each assessment year as well as the children’s mean ages in third, fourth, and fifth grade. A detailed sample description of keyage and OTK children, including mean test scores in third, fourth, and fifth grade for boys and girls is provided in Supplementary Tables S1, S2, and S3.

**Table 1:** Description of keyage and OTK children in third, fourth, and fifth grade.

	Group	
	Keyage children	OTK children
Grade 3		
*N* children	1118 (52% girls)	261 (39% girls)
*N* observations	6294	1469
*N* schools	33	34
Age *M (SD)*	8.53 (0.28)	9.40 (0.38)
Grade 4		
*N* children	1118 (51% girls)	279 (38% girls)
*N* observations	6474	1616
*N* schools	32	32
Age *M (SD)*	9.52 (0.29)	10.39 (0.37)
Grade 5		
*N* children	957 (52% girls)	236 (36% girls)
*N* observations	5426	1314
*N* schools	33	33
Age *M (SD)*	10.57 (0.28)	11.44 (0.37)
Total		
*N* children	1206 (51% girls)	296 (38% girls)
*N* observations	18,194	4399
*N* schools	34	35

### Physical fitness tests

The EMOTIKON test battery assesses the six physical fitness components cardiorespiratory endurance (i.e., 6-min run test), coordination (i.e., star-run test), speed (i.e., 20-m linear sprint test), lower (i.e., standing long jump test) and upper (i.e., ball-push test) limbs muscle power, and static balance (i.e., one-legged-stance test with eyes closed). The physical fitness tests were administered by physical education teachers, following a standard procedure (for more details see uni-potsdam.de/en/emotikon/projekt/methodik). Before test administration, children received warm-up exercises consisting of running exercises and games (e.g., playing tag). During the test instructions but not while performing the test, students were encouraged to achieve their best performance in the physical fitness tests. For tests with two trials, both trials were conducted on the same day.

#### Cardiorespiratory endurance

Children’s cardiorespiratory endurance was tested using the 6-min run test. Over a 6-minute time span, the participating children were asked to run the longest possible distance around a volleyball field measuring 9 m × 18 m = 54 m. The field was marked using six pylons that were set at a 9 m distance from each other. If a child stopped between two pylons at the stop signal, they were allowed to continue to the next pylon. The distance covered during the six minutes up to that last pylon was recorded in meters and used for further analysis. In children aged 7–11 years, the 6-min run test showed a test–retest reliability of *r* = 0.92^26^.

#### Coordination

The star-run test was used to assess coordination under time pressure. Children had to complete a star-shaped parkour with a total distance of 50.912 m and run as fast as possible. Four pylons marked the corners of a 9 m × 9 m square and one pylon marked the midpoint. Starting from the midpoint, children had to run to each of the other four pylons, touch it by hand and run back to the center. During the parkour, they had to use different movement directions and movement forms (i.e., running forward, running backward, side-steps to the left side, side-steps to the right side) in a standardized order. Each child completed the star-run test twice, the time was measured with a stopwatch with a 1/10 s accuracy. The score of the faster of the two trials was used for analysis. In children aged 8–10 years, the star-run test showed a test–retest reliability (intra-class correlation coefficient, ICC) of 0.68 (95% CI 0.53–0.79)^36^.

#### Speed

Linear sprint speed was tested using the 20-m sprint test. The children started the sprint from a standing upright position after an acoustic signal. Time was measured in seconds with a 1/10 s accuracy. The children had two trials; the fastest trial was used for analysis. The 20-m sprint test showed an excellent test–retest reliability of *r* = 0.90 in children aged 7–11 years^26^.

#### Lower limbs muscle power (PowerLOW)

The standing long jump test was used to assess muscle power of the lower limbs (PowerLOW). From a standing upright position, the participants were asked to jump as far as possible in a horizontal direction with their feet parallel and in a shoulder-width stance. They had to jump with both legs and land with both feet together. Children were allowed to swing their arms before and during the jump but they were not allowed to touch the floor with their hands after landing. The distance between their toes at take-off and their heels at landing was measured with a 1 cm accuracy. The children completed the standing-long-jump twice, the trial with the better jump distance was used for analysis. The standing long jump test showed excellent test–retest reliability (ICC) of 0.94 (95% CI 0.93–0.95) in children aged 6–12 years^37^.

#### Upper limbs muscle power (PowerUP)

The ball-push test was used to assess muscle power of the upper limbs (PowerUP). The children had to hold a 1 kg medicine ball in front of their chest with their arms bent and then push the ball as far as possible in a horizontal direction using both hands. The covered distance was measured in meters with a 10 cm accuracy. The children completed the ball-push test twice, and the best trial (largest distance) was used for analysis. In children aged 8 to 10 years, the ball-push test showed acceptable test–retest reliability (ICC) of 0.81 (95% CI 0.71–0.87)^36^.

#### Static balance

Children’s static balance was measured using the one-legged-stance test with eyes closed. The children stood with hands held akimbo, their standing leg slightly bent, both knees pointing forward, and the free leg bent between 60° and 90° at the hip joint and approximately 90° at the knee joint. This position was visually controlled by the physical education teacher. Thereafter, participants closed their eyes and remained in this position for as long as possible. Time was measured with a 1 s accuracy. A score of 60 s indicated excellent performance and the maximum duration of a trial was set at 60 s. Only if children’s test trial lasted less than five seconds, they were granted another trial. For the analyses, scores larger than 60 s, indicating that the test had not been ended in time, were set to the maximal value of 60 s. The one-legged-stance test with eyes closed showed acceptable test–retest reliability (ICC) of 0.69 (95% CI 0.61–0.75) in children aged 7–10 years^38^.

### Statistics

Preprocessing of data and data analysis was done in *R* (4.2.3)^39^, the *RStudio IDE*^40^, and in *Julia* (Version 1.9.0)^41^. For data preprocessing we used the suite of *tidyverse* packages^42^ and to specify contrasts the *Hypr*^43^ and *MASS*^44^ packages. Linear mixed effect models were performed using *lme4*^45^ in *R* and the *MixedModels* package^46^ in *Julia*.

Statistical analyses were similar to the ones reported in Fühner et al.^22,23^. A box-cox distributional analysis^47^ indicated that for the star-run and the 20-m sprint test, a reciprocal transformation and for the one-legged stance test a logarithmic transformation of the test scores were required for a normal distribution of model residuals.

The original unit of the star-run and the 20-m sprint test was seconds. We multiplied the reciprocal scores (1/s) of the star-run with 50.912 (distance in meters of the star-run) and the reciprocal scores of the 20-m sprint with 20 (distance in meters of the 20-m sprint), thus transforming their units into meters/seconds. Consequently, for all six tests, higher scores indicate a better performance.

Test scores were standardized (i.e., converted to z-scores). To identify outliers, we calculated z-scores separately for boys and girls for each test. For all tests, except for the one-legged-stance test, we excluded scores outside of a ± 3 *SD* range. The one-legged-stance test was terminated after a maximum of 60 s. Accordingly, test scores larger than 60 s were not possible. As the whole range of scores of the one-legged-stance test indicate valid performance, we did not apply the ± 3 *SD* criterion to this test. Then, in a second step, z-scores were recomputed separately for each test, aggregated over boys and girls to keep sex-related differences in the data.

For analyses, the six physical fitness tests were treated as six levels of the factor ‘physical fitness component’ (i.e., cardiorespiratory endurance, coordination, speed, powerLOW, powerUP, and balance). This was possible as test scores were standardized and allowed us to fit LMMs that took into account child- and school-related correlations between tests. Details regarding parsimonious model selection^48^ are reported in script *LS_lmm.qmd* in the OSF repository. The factor ‘group’ included six factor levels (i.e., keyage third-graders, keyage fourth-graders, keyage fifth-graders, and OTK third-graders, OTK fourth-graders, and OTK fifth-graders). Age was centered at 8.50 years for keyage third-graders, at 9.50 years for keyage fourth-graders, at 10.50 years for keyage fifth-graders; and centered at 9.50 years for OTK third-graders, at 10.50 years for OTK fourth-graders, and at 11.50 years for OTK fifth-graders. The final LMM included fixed effects for group, sex, and age, all nested under the six levels of the factor physical fitness component. The random effects structure of the selected LMM included random variance components (VCs) and correlation parameters (CPs) for the test scores for child (*N* = 1502) and school (*N* = 35). We chose a level of significance of |z|≥ 2.0.

Contrasts for physical fitness components were adopted from Fühner et al.^22,23^, that is, sequential difference contrasts compared coordination vs. cardiorespiratory endurance, speed vs. coordination, powerLOW vs. speed, and powerUP vs. powerLOW. Fühner et al.^22,23^ did not assess balance, so we added an additional fifth contrast that compared balance vs. powerUP. For the sex contrast (i.e., boys vs. girls), we used a sequential difference contrast with positive estimates indicating a better performance for boys, and negative estimates indicating a better performance for girls. For the six-level factor ‘group’, we specified five contrasts: (1) longitudinal development from third to fourth grade (i.e., aggregated over keyage and OTK children), (2) longitudinal development from fourth to fifth grade (i.e., aggregated over keyage and OTK children), (3) keyage fourth-graders vs. age-matched OTK third-graders, (4) keyage fifth-graders vs. age-matched OTK fourth-graders, and (5) keyage children vs. OTK children (i.e., aggregated over three assessment years, cross-sectional contrast). The third and fourth contrasts test the critical difference between keyage and OTK children at the same age (i.e., a cross-sectional comparison across different school grades).

Some research questions were addressed with inferences based on the estimation of LMM variance components and correlation parameters as well as additional post-hoc LMMs.

## Results

**Figure 1:**
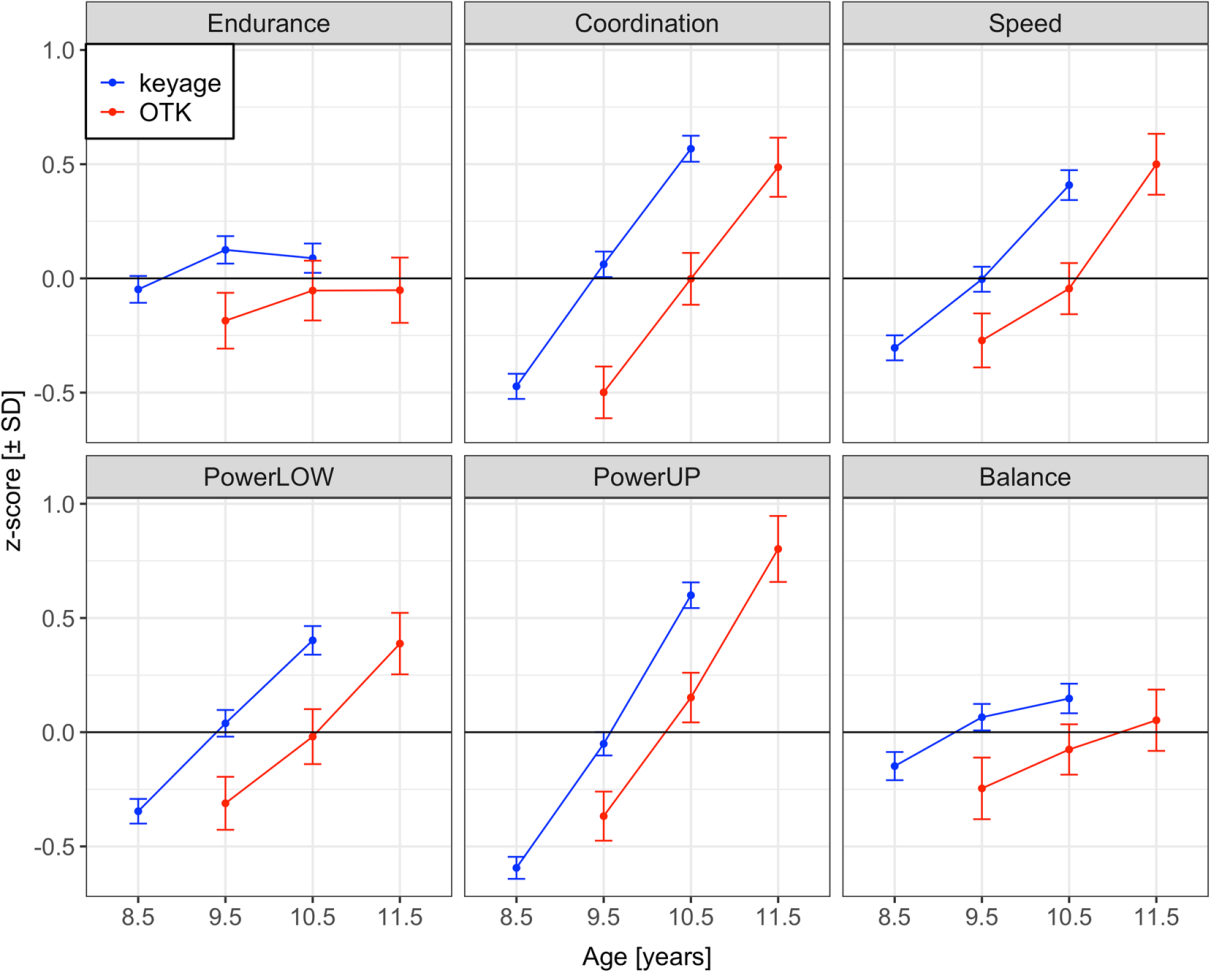
Performance (means and 95% CIs) in six physical fitness tests for keyage children (blue) and OTK children (red). The lines represent the physical fitness development from third to fourth, and from fourth to fifth grade. Keyage children were approximately 8.5 years in third, 9.5 years in fourth, and 10.5 years old in fifth grade, OTK children were approximately 9.5 years in third, 10.5 years in fourth, and 11.5 years old in fifth grade. Data were z-transformed. Endurance = cardiorespiratory endurance (i.e., 6-min-run test), Coordination = star-run test, Speed = 20-m linear sprint test, PowerLOW = lower limbs muscle power (i.e., standing long jump test), PowerUP = upper limbs muscle power (i.e., ball-push test), Balance = static balance (i.e., one-legged-stance test with eyes closed). For coordination and speed, scores were converted from seconds to meters/seconds (i.e., pace scores; star-run test = 50.912 [m]/time [s]; 20-m linear sprint test = 20 [m]/time [s]). For static balance, scores were log-transformed. For all physical fitness tests, a larger z-score indicates a better physical fitness.

Table 2 shows the fixed effects of the LMM, their standard error and z-values. Because z-scores are scores transformed in units of *SD*, the estimates of the LMM also indicate their effect size in *SD*-units. Figure 1 shows the performance for keyage and OTK children in third (i.e., age approximately 8.5 years for keyage and 9.5 years for OTK children), fourth (i.e., age approximately 9.5 years for keyage and 10.5 years for OTK children) and fifth grade (i.e., age approximately 10.5 years for keyage and 11.5 years for OTK children) in six physical fitness tests assessing cardiorespiratory endurance, coordination, speed, powerLOW, powerUP, and balance.

**Table 2:** Fixed effect estimates of the linear mixed model, standard errors, z-values and effects in test score units.

Source of variance	Fixed-effect estimate	Standard error	z-values	Effect in test score unit (b*SD)
Grand mean (intercept)	0.021	0.041	0.51	
Physical fitness component				
Coordination vs. endurance	0.042	0.084	0.50	
Speed vs. coordination	< 0.001	0.070	< 0.01	
PowerLOW vs. speed	− 0.005	0.059	− 0.08	
PowerUP vs. powerLOW	0.098	0.059	1.68	
Balance vs. powerUP	− 0.074	0.077	− 0.96	
Development from grade 3 to grade 4				
Endurance	0.166	0.032	**5.21**	26.6 m
Coordination	0.516	0.032	**16.36**	0.16 m/s
Speed	0.264	0.031	**8.50**	0.12 m/s
PowerLOW	0.334	0.032	**10.57**	7.3 cm
PowerUP	0.526	0.031	**16.78**	44 cm
Balance	0.198	0.032	**6.23**	0.18 log(s)
Development from grade 4 to grade 5				
Endurance	− 0.064	0.034	− 1.91	− 10.2 m
Coordination	0.503	0.033	**15.36**	0.16 m/s
Speed	0.446	0.033	**13.72**	0.21 m/s
PowerLOW	0.367	0.032	**11.29**	8.0 cm
PowerUP	0.620	0.033	**19.02**	52 cm
Balance	0.130	0.033	**3.92**	0.12 log(s)
Age-matched comparison 1: Keyage fourth-graders vs. OTK third-graders				
Endurance	0.341	0.063	**5.40**	54.6 m
Coordination	0.531	0.060	**8.80**	0.17 m/s
Speed	0.258	0.062	**4.14**	0.12 m/s
PowerLOW	0.350	0.066	**5.32**	7.6 cm
PowerUP	0.277	0.058	**4.75**	23 cm
Balance	0.241	0.060	**4.04**	0.22 log(s)
Age-matched comparison 2: Keyage fifth-graders vs. OTK fourth-graders				
Endurance	0.119	0.063	1.88	19.0 m
Coordination	0.555	0.060	**9.27**	0.18 m/s
Speed	0.406	0.062	**6.51**	0.19 m/s
PowerLOW	0.393	0.065	**6.04**	8.6 cm
PowerUP	0.384	0.058	**6.63**	32 cm
Balance	0.176	0.059	**2.97**	0.16 log(s)
Keyage children vs. OTK children (aggregated over assessment years)				
Endurance	0.174	0.051	**3.41**	27.8 m
Coordination	0.035	0.048	0.73	0.01 m/s
Speed	− 0.039	0.051	− 0.78	− 0.02 m/s
PowerLOW	0.013	0.054	0.24	0.28 cm
PowerUP	− 0.245	0.045	− **5.40**	− 21 cm
Balance	0.044	0.047	0.94	0.04 log(s)
Sex				
Endurance: sex	0.418	0.039	**10.64**	66.9 m
Coordination: sex	0.152	0.037	**4.13**	0.05 m/s
Speed: sex	0.218	0.039	**5.56**	0.10 m/s
PowerLOW: sex	0.317	0.042	**7.60**	6.9 cm
PowerUP: sex	0.530	0.035	**15.16**	45 cm
Balance: sex	− 0.353	0.036	− **9.86**	− 0.32 log(s)
Age (within-group, cross-sectional)				
Endurance: age	0.074	0.063	1.18	11.8 m
Coordination: age	0.157	0.059	**2.68**	0.05 m/s
Speed: age	0.164	0.062	**2.63**	0.08 m/s
PowerLOW: age	0.150	0.066	**2.26**	3.3 cm
PowerUP: age	0.449	0.056	**7.98**	38 cm
Balance: age	0.122	0.058	**2.12**	0.11 log(s)

Endurance = cardiorespiratory endurance (i.e., 6-min run test), coordination = star-run test, speed = 20-m linear sprint test, powerLOW = lower limbs muscle power (i.e., standing long jump test), powerUP = upper limbs muscle power (i.e., ball-push test), Balance = static balance (i.e., one-legged-stance with eyes closed). Bold =|z|> 2.0, linear mixed model random factors: schools (35) and children (1,502), observations = 22,593. For estimates of variance components and correlation parameters see Table 3.

### Longitudinal physical fitness development from third to fourth grade

For each of the six physical fitness components, the contrast comparing third-graders with fourth-graders was significant, meaning that in each fitness component, the children’s performance improved from third to fourth grade. The largest developmental gain was observed for the ball-push test assessing powerUP (*b* = 0.526, z = 16.78), where children pushed the medicine ball 44 cm further in fourth than in third grade. The smallest one-year fitness gain was found for the 6-min run assessing cardiorespiratory endurance (*b* = 0.166, z = 5.21), where fourth-graders ran 27 m further than third-graders. A post-hoc LMM showed no evidence for differences in the developmental rates of keyage and OTK children from third to fourth grade. This post-hoc LMM is reported in script *LS_lmm.qmd* in the OSF repository.

### Longitudinal physical fitness development from fourth to fifth grade

Children improved in five of six physical fitness tests from fourth to fifth grade. The exception was the 6-min run test, where the change from fourth to fifth grade was not significant. As was the case for development from third to fourth grade, the largest developmental one-year fitness gain was observed for the ball-push test assessing powerUP (*b* = 0.620, z = 19.02). This change translates to a 52 cm performance increase. The smallest significant one-year fitness gain was found for the one-legged-stance test assessing balance (*b* = 0.130, z = 3.92). A post-hoc LMM testing the group (i.e., keyage vs. OTK children) x grade (i.e., grade 4 vs. grade 5) interaction showed that OTK children exhibited a slightly larger developmental rate in the 20-m sprint test performance from fourth to fifth grade, compared to keyage children (*b* = − 0.131, z = − 2.03). For the other fitness tests, there was no evidence for differences in the developmental rates from fourth to fifth grade between keyage and OTK children. This post-hoc LMM is reported in script *LS_lmm.qmd* in the OSF repository.

### Age-matched comparison 1: Physical fitness of keyage fourth-graders vs. OTK third-graders

Contrasts comparing the performance of OTK third-graders with the performance of age-matched keyage fourth-graders (i.e., both groups approximately 9.5 years old) showed that for all six physical fitness components, keyage children outperformed OTK children. Interestingly, the largest difference was found for the star-run test assessing coordination (*b* = 0.531, z = 8.80), where keyage fourth-graders completed the star-run test 0.17 m/s (i.e., ≈ 2 s) faster than OTK third-graders. In the standing long jump test (*b* = 0.350, z = 5.32), keyage fourth-graders jumped approximately 8 cm further than OTK third-graders. Keyage fourth-graders outperformed OTK third-graders in the 6-min run test (*b* = 0.341, z = 5.40), where they ran approximately 55 m further. The group difference in the ball-push test (*b* = 0.277, z = 4.75) translates into a performance difference of 23 cm. The smallest group differences were found for the 20-m sprint test (*b* = 0.258, z = 4.14), were keyage fourth-graders ran 0.12 m/s (i.e., ≈ 0.1 s) faster than OTK third-graders, and for the one-legged-stance test (*b* = 0.241, z = 4.04).

### Age-matched comparison 2: Physical fitness of keyage fifth-graders vs. OTK fourth-graders

The contrasts comparing the performance of keyage fifth-graders with the performance of age-matched OTK fourth-graders (i.e., both groups approximately 10.5 years old) showed that in five out of six physical fitness tests, keyage children outperformed OTK children. Again, the largest group difference was found for the star-run test assessing coordination (*b* = 0.555, z = 9.27), where keyage fifth-graders completed the test 0.18 m/s (i.e., ≈ 2 s) faster than OTK fourth-graders. In the 20-m sprint test, keyage children ran approximately 0.19 m/s (i.e., ≈ 0.2 s) faster than OTK children (*b* = 0.406, z = 6.51). In the standing long jump test (*b* = 0.393, z = 6.04) and the ball-push test (*b* = 0.384, z = 6.63), keyage children jumped 9 cm longer and pushed the medicine ball 32 cm further than OTK children, respectively. The smallest group difference was observed for the one-legged-stance test (*b* = 0.176, z = 2.97). Interestingly, there was no evidence for a performance difference in the 6-min run test between keyage fifth-graders and OTK fourth-graders (z < 2).

As shown in Fig. 1, keyage and OTK children seem to exhibit different developmental rates between the ages 9.5 and 10.5 years in some fitness components, most visible in the 6-min run test. A post-hoc LMM testing whether development between these ages differed between keyage and OTK children revealed two significant interactions: For the 6-min run test, OTK children exhibited a larger developmental gain, whereas for the 20-m sprint test, keyage children exhibited a slightly larger developmental gain between the ages 9.5 and 10.5 years. This post-hoc LMM is reported in script *LS_lmm.qmd* in the OSF repository.

### Keyage vs. OTK children aggregated over three assessment years

The next group of contrasts compared keyage children with OTK children when performance was aggregated over three assessment years (i.e., in this comparison, OTK children were compared to keyage children who were on average one year younger). Keyage children showed better 6-min run test performance compared to OTK children who were on average one year older (*b* = 0.174, z = 3.41). OTK children exhibited a better ball-push test performance compared to keyage children (*b* = − 0.245, z = − 5.40). In four out of six fitness tests (i.e., star-run test, 20-m sprint test, standing long jump test, and one-legged-stance test), there was no evidence for a difference between OTK children and keyage children when performance was aggregated over all three school grades.

### Sex differences in physical fitness

Boys outperformed girls in five of six physical fitness tests (*b*s between 0.152 for the star-run test and 0.530 for the ball-push test, z-values between 4.13 for the star-run test and 15.16 for the ball-push test). The exception was the one-legged-stance test, with girls performing significantly better than boys (*b* = − 0.353, z = − 9.86).

#### Cross-sectional within-group age effects on physical fitness

Within each group of children (i.e., keyage third-graders, keyage fourth-graders, keyage fifth-graders, OTK third-graders, OTK fourth-graders, OTK fifth-graders), the effect of age relative to the mean age of the six groups was significant for five of six fitness tests (i.e., star-run test, 20-m sprint test, standing long jump test, ball-push test, and one-legged-stance test) with relatively older children outperforming relatively younger children. The largest cross-sectional age effect was observed for the ball-push test (*b* = 0.449, z = 7.98), followed by the 20-m sprint test (*b* = 0.164, z = 2.63), the star-run test (*b* = 0.157, z = 2.68), the standing-long-jump test (*b* = 0.150, z = 2.26), and the one-legged-stance test (*b* = 0.122, z = 2.12). For the 6-min run test, there was no evidence for a cross-sectional within-group age effect (z < 2).

Table 3 shows the variance components and correlation parameters of the random effects structure. Individual differences between children (0.29–0.48) were larger than differences between schools (0.07–0.14). For children, performance in the 6-min run test, star-run, 20-m sprint test, and standing-long jump test (i.e., assessing cardiorespiratory endurance, coordination, speed and powerLOW) correlated highly with each other (0.77–0.98)—clearly representing a latent construct of “physical fitness”. Correlations of the ball-push test and the one-legged-stance test with the other four tests are much smaller (0.23–0.60). Correlation parameters for schools are smaller and less systematic than those for children.

**Table 3:** Variance components and correlation parameters of test scores.

Fitness component	VC	CP					
		Endurance	Coordination	Speed	PowerLOW	PowerUP	Balance
Child							
Endurance	0.40	1.00					
Coordination	0.33	0.87	1.00				
Speed	0.41	0.86	0.96	1.00			
PowerLOW	0.48	0.77	0.91	0.98	1.00		
PowerUP	0.29	0.23	0.60	0.52	0.58	1.00	
Balance	0.31	0.36	0.45	0.41	0.40	0.14	1.00
School							
Endurance	0.14	1.00					
Coordination	0.14	0.21	1.00				
Speed	0.11	0.52	0.39	1.00			
PowerLOW	0.10	0.54	0.54	0.51	1.00		
PowerUP	0.07	0.20	0.53	0.33	0.48	1.00	
Balance	0.13	0.09	0.30	− 0.28	0.09	0.14	1.00

Endurance = cardiorespiratory endurance (i.e., 6-min run test), Coordination = star-run test, Speed = 20-m linear sprint test, PowerLOW = lower limbs muscle power (i.e., standing long jump test), PowerUP = upper limbs muscle power (i.e., ball-push test), static balance (i.e., one-legged-stance with eyes closed). *VC* variance component, *CP* correlation parameter. VC for residual = 0.40.

### Post-hoc exploratory analyses

#### Signal of puberty: age × sex interactions

Previous research has shown that within the ninth year of life, boys outperform girls in most fitness tests, but developmental rates are completely linear and statistically parallel for both sexes, showing no signal of earlier puberty of girls^22^. As the present study tested children over a longer time period up until the twelfth year of life, and as pubertal growth starts approximately two years earlier in girls than in boys^33^, we tested whether we would pick up such a signal of puberty. In two exploratory post-hoc LMMs, one for keyage and one for OTK children, we tested whether girls and boys exhibited different developmental rates from third to fourth and from fourth to fifth grade. Details on these LMMs can be found in script *LS_lmm.qmd* in the OSF repository. Keyage children, who were only tested up until their eleventh year of life in the present study, exhibited no evidence for sex x school grade interactions in any physical fitness component. OTK children, on the other hand, were one year older than keyage children and mostly in their twelfth year of life in fifth grade. For OTK children, we indeed found a sex x school grade interaction in two fitness tests: from fourth to fifth grade, OTK girls exhibited a larger developmental rate in the 20-m sprint and standing long jump test than OTK boys. This is shown in Fig. S1 in the supplements. Performances in the 20-m sprint test and the standing long jump test are highly correlated (i.e., see Table 3), presumably because both tests require lower limbs muscle power.

#### Control of retest effects

Our analyses compared keyage children with age-matched OTK children (i.e., keyage fourth-graders vs. OTK third-graders, and keyage fifth-graders vs. OTK fourth-graders), meaning that in these comparisons, keyage children were one school grade ahead of OTK children. Due to the longitudinal nature of this study that included a first assessment in third grade and a second and third assessment in grades four and five, respectively, we tested whether retest effects were responsible for the better performance of keyage children when compared to age-matched OTK children. We conducted two post-hoc LMMs, one for fourth-graders, and one for fifth-graders. Details on these LMMs can be found in script *LS_lmm.qmd* in the OSF repository. In the first model, we compared fourth-graders with two assessments (i.e., those who had already participated in third grade) with fourth-graders who had *not* participated previously in third-grade. Note that children with only one assessment during the three-year study period were not included in the main analyses of this study. To control for school-related differences in the children’s physical fitness, we only kept children with or without a retest from the same schools. This left us with 1,190 fourth-graders with a retest and 114 new fourth-graders from 29 schools. There was no evidence for a retest effect on any of the physical fitness components (|z-values|< 1.5). In the second LMM, we compared fifth-graders with three assessments (i.e., who had already participated in third and in fourth grade, *N* = 818) with fifth-graders with one or two assessments (*N* = 264) from 26 schools. There were two significant retest effects for the 6-min run test (*b* = 0.253, *z* = 3.61) and for the star-run test (*b* = 0.147, z = 2.40). Despite the retest effect for the 6-min run test, there was no significant difference in the 6-min run performance between keyage fifth-graders compared to OTK fourth-graders in the main LMM reported in the present study. This retest effect can thus be neglected. For the star-run test, the estimate in the main LMM comparing keyage fifth-graders to OTK fourth-graders was almost four times as large as the retest effect (0.555 > 0.147). Our results therefore indicate real group differences, not retest effects.

#### Distribution of child-related conditional means

**Figure 2:**
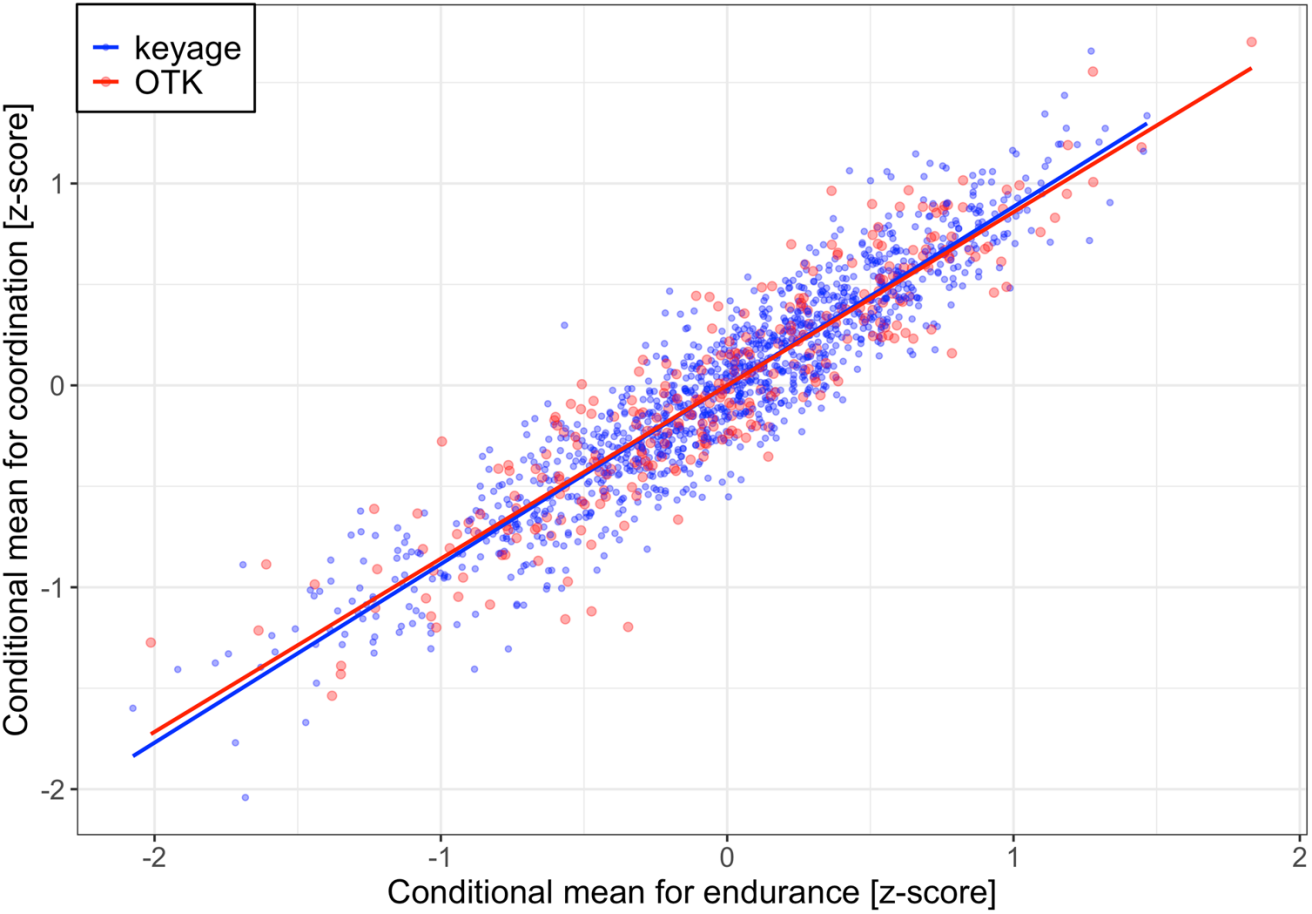
Correlation between the conditional means for cardiorespiratory endurance (i.e., 6-min run-test) and coordination (i.e., star-run test) for keyage children (blue) and OTK children (red).

Child-related conditional means of the tests may reveal an additional clustering of OTK children. For example, a subgroup of OTK children that initially might have been developmentally delayed at school entry might have compensated their developmental delay during the first two school grades. Some OTK children might also have been held back at the wish of parents, not based on evidence from the school entry examination. We do not have such information about the children, but, in principle, individual differences related to these hypotheses might appear as distinct clusters of OTK children in one or several scatterplot panels. Figure 2 shows the correlation between the conditional means for the 6-min run test (i.e., assessing cardiorespiratory endurance) and the conditional means for the star-run test (i.e., assessing coordination) for keyage and OTK children. There is no evidence in support of this hypothesis. In fact, OTK children appear to be perfectly mixed with keyage children, suggesting that the group-related fixed effects adequately absorbed all of the relevant variance. Additional scatterplot panels showing similar results for the other physical fitness tests for keyage and OTK children are documented in script *LS_lmm_fig.Rmd* in the OSF repository.

#### Hypothesis: OTK children are chronologically older, but biologically younger (less mature) than keyage children

The results are in agreement with the hypothesis that OTK children exhibited lower physical fitness compared to age-matched keyage children. One possible reason for their lower physical fitness could be that some OTK children are biologically younger than indicated by their chronological age. Specifically, at the school entry examination, OTK children may have been judged to be not mature enough (i.e., biologically younger) and consequently held back for one year. This selection-by-judged-maturity effect implies that the *chronologically older* OTK children are likely the *biologically younger* OTK children. Keyage children of the same chronological age also vary in their biological age, but, in the absence of such a selection bias for them, we may assume that biological age is normally distributed around each chronological age. Consequently, on average (i.e., at the group level) there is no difference between chronological and biological age for keyage children. Children’s biological age can be estimated using anthropometric data^49,50^ or hand-wrist radiographs^51^. As this information was not available for the children in this study, we propose a simple formula to transform OTK children’s chronological age into a proxy for their biological age. Specifically, the formula *assumed biological age* = *9 years − (key date − birthdate)/365* subtracts the interval between birthdate and key date in the school enrollment year from the age of 9 years. As the OTK children’s age is ‘mirrored’ at 9 years, the chronologically oldest OTK become the biologically youngest OTK and the chronologically youngest OTK become the biologically oldest OTK children.

#### Transforming OTK children’s chronological age into a proxy for biological age

**Figure 3:**
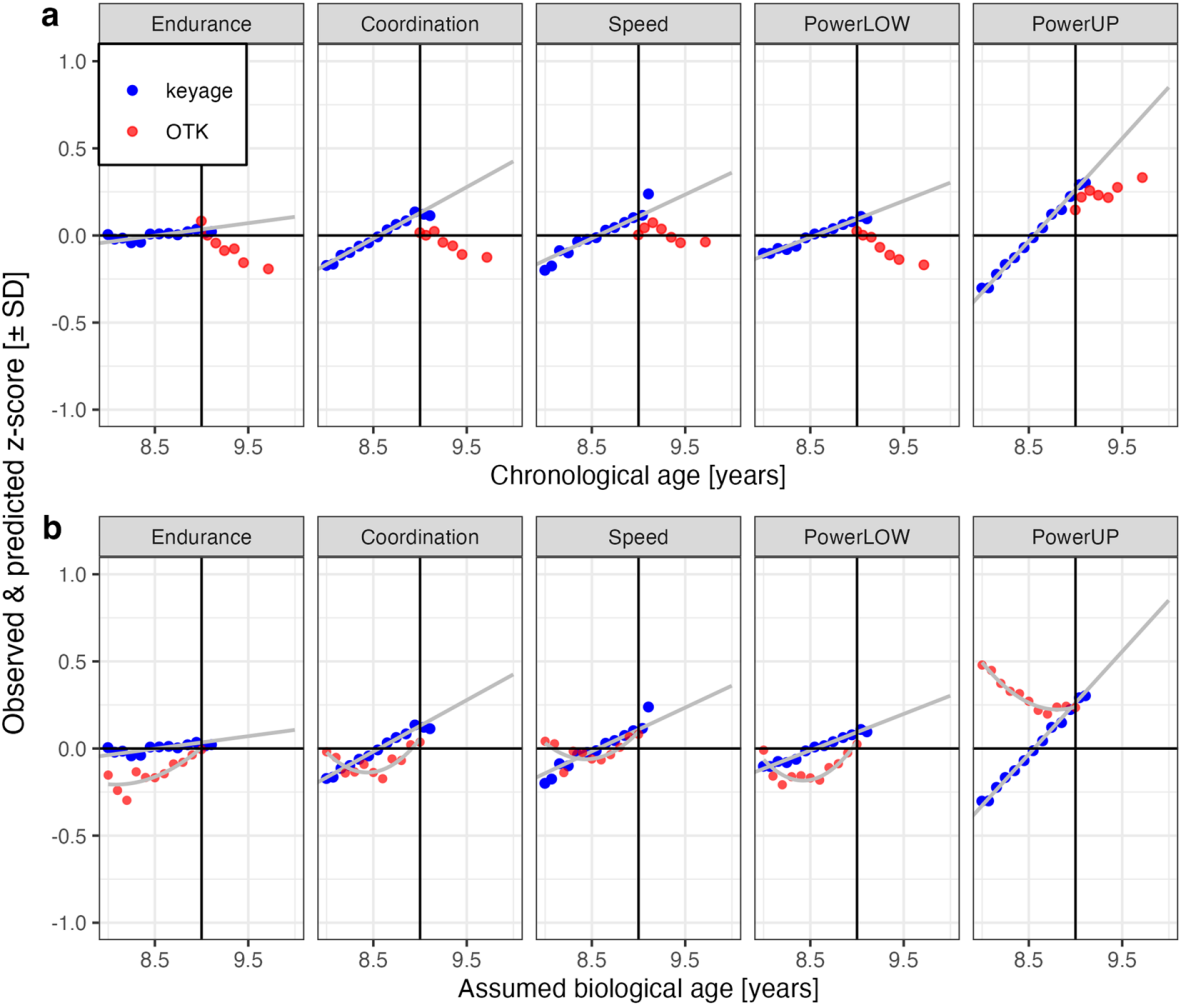
Cross-sectional development in five physical fitness components for keyage children (blue symbols) and OTK children (red symbols). The horizontal line marks 9.0 years (i.e., typical age boundary between keyage and OTK third-graders). Panel (**a**) shows the cross-sectional developmental rates according to the chronological age. Panel (**b**) shows the cross-sectional developmental rates according to the proxy for biological age. Data come from 108,295 keyage and 26,540 OTK third-graders reported in Fühner et al.^23^ and were z-transformed. Points are binned child means. Z-scores for children between the ages 7.0 and 8.0 years and for children between 9.5 and 10.0 years were aggregated. Endurance = cardiorespiratory endurance (i.e., 6-min-run test), Coordination = star-run test, Speed = 20-m linear sprint test, PowerLOW = lower limbs muscle power (i.e., standing long jump test), PowerUP = upper limbs muscle power (i.e., ball-push test). For coordination and speed, scores were converted from seconds to meters/seconds (i.e., pace scores; star-run test = 50.912 [m]/time [s]; 20-m linear sprint test = 20 [m]/time [s]). For all physical fitness tests, a larger z-score indicates a better physical fitness.

We tested whether our formula transforming OTK children’s chronological age into a proxy for their biological age would bring their cross-sectional age-related development in line with the age-related development of keyage children. To test this transformation of the OTK children’s chronological age, we reanalyzed the physical fitness data of OTK third-graders (*N* = 26,540) reported in a previous study^23^. Panel (a) of Fig. 3 (adapted from Fühner et al.^23^) shows the cross-sectional development for five physical fitness components of keyage and OTK children for chronological age. OTK children exhibit an initial cross-sectional physical fitness decline with increasing age, followed by a plateau for older OTK children for cardiorespiratory endurance, coordination, speed and powerLOW. Panel (b) of Fig. 3 shows the cross-sectional development using children’s “biological” age computed with the formula described above. For “biologically” older OTK children (i.e., assumed biological age between 8.5 and 9.0 years), the development is linearly positive and of a similar rate for the same four physical fitness tests. The exception in both panels is powerUP, where most OTK children outperform keyage children of the same assumed biological age, and only the biologically older OTK children show a positive linear development similar to keyage children. The transformation does not fully bring the developmental rate of OTK children in line with the developmental rate of keyage children. Thus, the mapping of chronological and biological age of OTK children requires further research. However, the analysis is consistent with the hypothesis that biological age might be a factor associated with the lower physical fitness of OTK children, especially for the biologically older/chronologically younger ones.

## Discussion

In this longitudinal study, we examined the effect of timing of school enrollment on six physical fitness components (i.e., cardiorespiratory endurance, coordination, speed, muscle power of lower and upper limbs, and static balance) in primary school children. We assessed the physical fitness of 1,502 children in third, fourth, and fifth grade. Children included in the sample were tested in at least two assessment years. The main results of our study are: (1) Keyage children outperformed *age-matched* OTK children: when both groups of children were approximately 9.5 years old, keyage children exhibited better performance than OTK children in all six physical fitness components. One year later, when both groups were on average 10.5 years old, the fitness impairment of OTK children persisted, except for cardiorespiratory endurance, where no group difference was observed. (2) Both, keyage and OTK children, showed a positive one-year development in most physical fitness tests from third to fourth grade, and from fourth to fifth grade. The exception was cardiorespiratory endurance, which did not improve from fourth to fifth grade in either group of children. We will return to this result later in the discussion. (3) When aggregating performance over all three assessments and comparing OTK children with keyage children who were on average one year younger, keyage children outperformed OTK children in cardiorespiratory endurance, and OTK children outperformed keyage children in powerUP. In the other physical fitness components, there was no evidence that OTK children’s performance differed from the performance of keyage children who were on average one year younger.

Recently, Fühner et al.^23^ reported lower physical fitness of OTK third-graders compared to their predicted performance in five fitness components (i.e., cardiorespiratory endurance, coordination, speed, muscle power of lower and upper limbs; they did not assess balance). However, in their cross-sectional study including children from the same school grade, age and timing of school enrollment were confounded. With the longitudinal design employed here, we were able to compare age-matched children from different school grades and to examine the longitudinal development of both groups of children. This ultimately allowed us to dissociate effects of timing of school enrollment and age on physical fitness. Our results validate previous findings^23^, confirming that OTK children exhibit a delayed physical fitness development.

Moreover, as we examined children of a wider age range, results showed that the poorer physical fitness of OTK children compared to age-matched keyage children persisted until the eleventh year of life. The fitness impairments were thus not due to a temporary delay in third grade that could have been compensated with accelerated development in fourth or fifth grade. A post-hoc LMM comparing the developmental rates of keyage and OTK children from third to fifth grade revealed that both groups of children exhibited largely the same developmental rates during this time. There was only one significant interaction, with OTK children exhibiting a slightly larger development in speed from fourth to fifth grade than keyage children. This may be a signal of beginning puberty, as OTK children are one year older in fifth grade than keyage children. These results indicate that despite OTK children’s fitness deficits, they do not seem to further fall behind over time (but also do not catch up in most fitness components). Of course, OTK children’s physical fitness impairments may still diminish or increase in adolescence and adulthood. Interestingly, at both times of comparison (i.e., at 9.5 and 10.5 years of age), the largest group difference between keyage and OTK children was found for coordination. Coordination was tested by the star-run test, in which children had to memorize a star-like pattern associated with different directions and forms of movement. This test is associated with a higher cognitive load than the other fitness tests and not only requires physical fitness, but also working memory. The OTK children’s impairment in coordination is in line with previous research reporting academic deficits in reading or mathematics^29–32^.

Interestingly, OTK girls exhibited larger developmental rates than OTK boys in the 20-m sprint and the standing long jump test from fourth to fifth grade (i.e., average ages 10.5 and 11.5 years). Both tests require lower limbs muscle power and are highly correlated (i.e., see Table 3 in the results section). Pubertal growth starts approximately two years earlier in girls than in boys^33^. Their larger developmental gains in 20-m sprint and standing long jump test performance between the average ages 10.5 and 11.5 years likely reflect the earlier growth spurt and consequent increases in leg-length to sitting height ratio in girls^49^, as well as increases in muscle size and strength, associated with the rise of growth-related hormones during puberty^52^. Increases in the 20-m sprint test performance may also reflect better neuro-muscular control during maturation^53^. These results are in line with previous research showing that body height of children and adolescents is positively associated with performance in tests assessing speed and lower limbs muscle power^54–57^.

An unexpected result was that the 6-min run test performance (i.e., assessing cardiorespiratory endurance) did not improve after fourth grade, neither in keyage, nor in OTK children. In two large studies including European^58^ and German^59^ children and adolescents, cardiorespiratory endurance (i.e., tested with the 20-m shuttle run test and a static bicycle ergometer test, respectively) increased until it stabilized at around 14 to 15 years in girls and at around 15 to 16 years in boys, while a longitudinal study with German children aged 9 to 12 years reported a plateau between 11 and 12 years in the 9-min run test performance^60^. Possibly, the stagnation of cardiorespiratory endurance in our sample was due to motivational, rather than physical aspects, especially since performance in other running tests improved after fourth grade. The 6-min run is the longest test of the EMOTIKON test battery and children in fifth grade may have been less motivated to carry out the test with continuous effort. Interestingly, OTK children seemed to catch up to keyage children at an age of 10.5 years, as performance in the 6-min test run did not differ significantly between the groups at that age. However, as 10.5-year-old OTK *fourth-graders* were compared to 10.5-year-old keyage *fifth-graders,* the apparent ‘catch-up’ of OTK children at the age of 10.5 years relative to age-matched keyage children could also be interpreted as a consequence of the stagnation of endurance in keyage fifth-graders. OTK children also exhibited a stagnation of endurance in fifth grade, but consequently at an older age than keyage children.

Longitudinal development does not necessarily agree with cross-sectional developmental differences. In the present study, we tested both the longitudinal development, as well as cross-sectional within-group age effects (i.e., within the six groups of keyage children in third, fourth, and fifth grade, and OTK children in third, fourth, and fifth grade). Longitudinal age gains were larger than within-group cross-sectional age gains. Longitudinal development not only reflects ontogenetic development, but also environmental influences, like the effect of longer exposure to structured exercise in physical education classes. The longitudinal age gains from third to fourth grade and from fourth to fifth grade in the present study were largest for powerUP and coordination. The large age gain in powerUP is likely driven by age-related increases in body and muscle mass as well as body height^52,61,62^. Increases in coordination (i.e., star-run test performance), on the other hand, indicate increases in physical fitness as well as in executive function as children advance in school grades. Cross-sectional within-group age gains were also largest for powerUP. This was followed by speed, coordination, powerLOW, balance, and cardiorespiratory endurance, although the cross-sectional age gain for cardiorespiratory endurance was not significant. Note that the detection of cross-sectional age gains within one year of life is critically dependent upon sample size. In a large sample of 108,295 keyage third-graders between the ages 8.0 and 9.0 years, cross-sectional age gains were significant for all fitness components, including cardiorespiratory endurance^22^, whereas in a smaller study including 240 children, none of the cross-sectional age effects within the tenth year of life were significant^22,60^.

This study is not without limitations. When conducting longitudinal studies, retest effects need to be considered. We tested the possibility of a retest effect as the reason for the better performance of keyage fourth-graders compared to OTK third-graders (no evidence of retest effects), and for the better performance of keyage fifth-graders compared to OTK fourth-graders. For fifth-graders, there was evidence of two retest effects, one in cardiorespiratory endurance and in coordination. However, in the main analyses of this paper, there was no group difference between keyage fifth-graders and OTK fourth-graders in endurance, thus, this retest effect can be neglected. The retest effect for coordination was almost four times smaller than the corresponding estimate in the main analyses, showing that our results likely indicate reliable group differences.

Another limitation is related to the design employed in the present study. Comparing age-matched children across different school grades dissociates effects of ‘age’ and ‘time of school enrollment’ that are confounded in within-grade cross-sectional comparisons, but introduces another confound between ‘time of school enrollment’ and ‘number of years of physical education class’. As keyage children are always one grade ahead of age-matched OTK children, they have received one more year of structured exercise in physical education class than OTK children. Physical education programs can positively affect children’s cardiorespiratory endurance, muscular fitness, and measures of speed agility^63^ and may be considered as one reason for the better fitness of keyage children. However, this does neither explain the cross-sectional age-related decline of OTK children’s physical fitness in third grade^23^, nor the lower fitness of OTK children compared to their younger peers in the same grade^24^, as in these comparisons, all children were exposed to the same amount of structured exercise in physical education classes. Another difference between keyage and OTK children is that keyage children, due to their earlier school enrollment, have started to receive school-based physical education classes earlier in life than OTK children (i.e., keyage children received physical education classes starting in their seventh year of life, while OTK children entered school one year later). Early participation in exercise programs can enhance motor skill development in childhood^64,65^ and may be one factor associated with the better physical fitness of keyage compared to OTK children. The dissociation of age effects and effects of life events or so-called ‘period effects’ (i.e., in our case, exposure to physical education class can be interpreted as a period effect), is a common challenge in developmental science. For an in-depth discussion of the topic, we refer to early reports by Baltes^66^ and Schaie^67^, as well as a more recent review^68^. In our case, one way of dealing with this methodological challenge (while it may not be a very realistic option) could be to take advantage of the teacher shortage in Germany and to compare keyage fifth-graders who have only started receiving physical education classes in second grade with age-matched OTK fourth-graders who have started receiving physical education classes in first grade. This way, exposure to physical education classes would have started *at the same age* for both groups of children, and both groups of children would have been exposed to the *same amount* of school-based physical education classes at the time of assessment.

Children’s weight status affects their physical fitness^5^. As we only had information on the height and weight of some, but not all children, we were unable to compute all children’s body mass indices. However, the available anthropometric information yielded no systematic evidence for differences in weight status between keyage and OTK children (details can be found in script *LS_BMI.Rmd* in the OSF repository). The lack of skeletal age data using hand-wrist radiographs or at least anthropometric data (standing and sitting height) in the present study also meant that we were unable to estimate the children’s biological ages. Biological maturation affects children’s physical fitness and children of the same chronological age can largely vary in their biological age^27,69^. Moreover, we do not know the reasons for delayed school enrollment or repetition of a school year for the individual OTK children. In fact, it is likely that there are different groups of OTK children: OTK children whose parents voluntarily chose to delay their child’s school enrollment, even though their child did not exhibit developmental delays (voluntarily-delayed OTK), and OTK children who were held back or repeated a school year due to developmental delays (developmentally-delayed OTK). Developmental delays may simply be the result of a younger biological age, or might be the result of a lack of access to sports activities and associated with the children’s socioeconomic living environments. For instance, there is evidence that lower family socioeconomic status and higher socioeconomic deprivation of the school district increases the odds for global developmental delays in school entry examination^28^. We dealt with the uncertainties regarding the cause of the delayed school entry using two post-hoc exploratory analyses. First, we tested whether correlational patterns between the physical fitness components after group differences for the tests had been removed would indicate different subgroups of OTK children. However, the correlation patterns of OTK and keyage children did not differ and no clusters indicated OTK subgroups. Second, using data from a previous study with very large samples of keyage and OTK children^23^, we tested whether transforming OTK children’s chronological age into a proxy for their biological age by reflecting their chronological age at the legal key date would bring their developmental rate in line with the development of keyage children. Our formula was based on the assumption that OTK children are exposed to a selection-by-maturity effect, in which the chronologically youngest likely are the biologically oldest OTK children and the chronologically oldest are the biologically youngest OTK children. This transformation of chronological into a hypothetical proxy of biological age resulted in a positive linear fitness development for the biologically older OTK children (i.e., assumed biological age ranging from 9.0 down to 8.5 years; chronological age between 9.0 and 9.5 years). This function was similar to the one of keyage children, but did not result in a complete mapping of the developmental rates of OTK and keyage children. Especially chronologically older/biologically younger OTK children (i.e., assumed biological age ranging from 8.5 down to 8.0 years; chronological age between 9.5 and 10.0 years) exhibited large deviations from the developmental rate of keyage children. Skeletal age or anthropometric data required to estimate biological age^49,50^ would be needed to parameterize our zero-parameter formula employed here for a mapping of OTK children's chronological age to their biological age—not only to explain the relationship between time of school enrollment and physical fitness, but also to assess its effects in other educational contexts, like mathematics and language.

Our results show that OTK children indeed exhibit poorer physical fitness compared to age-matched keyage children, and that these deficits persist until the eleventh year of life. These results could have practical implications for physical education classes and other contexts in which grading is based on age norms. In light of the OTK children’s differences from keyage children of the same chronological age, grading of children’s performance might consider to put more weight on a positive intraindividual development of physical fitness and not solely focus on interindividual performance differences.

Results from the present study, together with previous research on cognitive abilities^29–32^ and physical fitness^23,24^, suggest that OTK children may not be impaired in single developmental areas, but may exhibit a more global developmental delay. As children’s physical fitness is related to various health-related outcomes^3^, OTK children may be more vulnerable to health issues in childhood and later in life. Future studies are needed to examine the cause of their developmental delays and to follow-up their development in adolescence and adulthood. A regular assessment of all children’s physical fitness can identify late physical fitness developers and assign them to intervention programs with the goal to promote their fitness. Interventions in the form of special attention during regular physical education classes, school-based or afterschool programs^70,71^ could be considered.

## Supplementary Information

Supplementary Information.

## Data Availability

Data as well as R and Julia scripts are available in the Open Science Framework (OSF) repository: https://osf.io/pemj6/.
