# Supplementary material for: Physical fitness of primary school children differs depending on their timing of school enrollment
Source: Sci Rep. 2023 May 31;13:8788. doi: 10.1038/s41598-023-35727-y (PMC10232550; doi:10.1038/s41598-023-35727-y)
Supplement: Supplementary file 1 — Supplementary Information. [file 41598_2023_35727_MOESM1_ESM.pdf]

**Physical fitness of primary school children differs depending on their timing of school enrollment**

Paula Teich, Thea Führner, Urs Granacher, Reinhold Kliegl

# Supplementary Material

**Table S1** Sample description of keyage and OTK children in third grade.

| *Group* | *Component* | *Sex* | *N Schools* | *N Children* | *Age (M)* | *Age (SD)* | *Score (M)* | *Score (SD)* |
|---|---|---|---|---|---|---|---|---|
| Keyage | Endurance | Boys | 33 | 505 | 8.54 | 0.27 | 1060.2 m | 166.3 m |
| Keyage | Endurance | Girls | 33 | 549 | 8.52 | 0.29 | 995.9 m | 139.6 m |
| Keyage | Coordination | Boys | 32 | 492 | 8.54 | 0.27 | 2.06 m/s | 0.31 m/s |
| Keyage | Coordination | Girls | 32 | 531 | 8.52 | 0.29 | 2.02 m/s | 0.27 m/s |
| Keyage | Speed | Boys | 33 | 520 | 8.54 | 0.27 | 4.65 m/s | 0.46 m/s |
| Keyage | Speed | Girls | 33 | 562 | 8.52 | 0.29 | 4.57 m/s | 0.41 m/s |
| Keyage | PowerLOW | Boys | 32 | 504 | 8.54 | 0.27 | 130.3 cm | 19.9 cm |
| Keyage | PowerLOW | Girls | 32 | 537 | 8.52 | 0.29 | 124.4 cm | 18.4 cm |
| Keyage | PowerUP | Boys | 32 | 513 | 8.54 | 0.28 | 3.97 m | 0.66 m |
| Keyage | PowerUP | Girls | 32 | 540 | 8.52 | 0.29 | 3.51 m | 0.62 m |
| Keyage | Balance | Boys | 32 | 504 | 8.53 | 0.28 | 16.1 s | 15.2 s |
| Keyage | Balance | Girls | 32 | 537 | 8.52 | 0.29 | 21.3 s | 18.1 s |
| OTK | Endurance | Boys | 34 | 146 | 9.34 | 0.34 | 1041.8 m | 151.68 m |
| OTK | Endurance | Girls | 28 | 95 | 9.48 | 0.42 | 947.6 m | 144.5 m |
| OTK | Coordination | Boys | 32 | 149 | 9.37 | 0.37 | 2.06 m/s | 0.29 m/s |
| OTK | Coordination | Girls | 28 | 94 | 9.48 | 0.41 | 1.99 m/s | 0.28 m/s |
| OTK | Speed | Boys | 34 | 157 | 9.35 | 0.36 | 4.69 m/s | 0.44 m/s |
| OTK | Speed | Girls | 27 | 95 | 9.49 | 0.41 | 4.51 m/s | 0.45 m/s |
| OTK | PowerLOW | Boys | 33 | 148 | 9.34 | 0.36 | 132.5 cm | 19.4 cm |
| OTK | PowerLOW | Girls | 27 | 92 | 9.47 | 0.41 | 120.7 cm | 18.9 cm |
| OTK | PowerUP | Boys | 33 | 154 | 9.34 | 0.36 | 4.09 m | 0.76 m |
| OTK | PowerUP | Girls | 27 | 95 | 9.48 | 0.41 | 3.67 m | 0.62 m |
| OTK | Balance | Boys | 33 | 148 | 9.35 | 0.36 | 16.6 s | 15.1 s |
| OTK | Balance | Girls | 27 | 96 | 9.47 | 0.41 | 19.1 s | 17.0 s |

Keyage = Children with school enrollment according to key date, OTK = Older-than-keyage children (i.e., delayed school enrollment or repetition of a grade). Component = Physical fitness component. Endurance = cardiorespiratory endurance (i.e., 6-min run test), coordination = star-run test, speed = 20-m linear sprint test, powerLOW = lower limbs muscle power (i.e., standing long jump test), powerUP = upper limbs muscle power (i.e., ball push test), Balance = one-legged-stance test with eyes closed. Score (*M*) = Mean test score, Score (*SD*) = Standard deviation of test scores.

**Table S2** Sample description of keyage and OTK children in fourth grade.

| *Group* | *Component* | *Sex* | *N Schools* | *N Children* | *Age (M)* | *Age (SD)* | *Score (M)* | *Score (SD)* |
|---|---|---|---|---|---|---|---|---|
| Keyage | Endurance | Boys | 32 | 511 | 9.53 | 0.28 | 1087.8 m | 172.8 m |
| Keyage | Endurance | Girls | 32 | 546 | 9.51 | 0.30 | 1023.5 m | 141.2 m |
| Keyage | Coordination | Boys | 32 | 508 | 9.53 | 0.28 | 2.22 m/s | 0.31 m/s |
| Keyage | Coordination | Girls | 32 | 540 | 9.51 | 0.29 | 2.19 m/s | 0.28 m/s |
| Keyage | Speed | Boys | 32 | 536 | 9.53 | 0.28 | 4.79 m/s | 0.46 m/s |
| Keyage | Speed | Girls | 32 | 562 | 9.52 | 0.30 | 4.71 m/s | 0.42 m/s |
| Keyage | PowerLOW | Boys | 32 | 531 | 9.53 | 0.28 | 138.5 cm | 22.8 cm |
| Keyage | PowerLOW | Girls | 32 | 562 | 9.52 | 0.29 | 132.9 cm | 19.8 cm |
| Keyage | PowerUP | Boys | 32 | 529 | 9.53 | 0.28 | 4.43 m | 0.72 m |
| Keyage | PowerUP | Girls | 32 | 558 | 9.52 | 0.29 | 3.97 m | 0.67 m |
| Keyage | Balance | Boys | 32 | 533 | 9.53 | 0.28 | 18.3 s | 16.1 s |
| Keyage | Balance | Girls | 32 | 558 | 9.52 | 0.30 | 25.4 s | 19.2 s |
| OTK | Endurance | Boys | 32 | 166 | 10.34 | 0.35 | 1056.0 m | 178.8 m |
| OTK | Endurance | Girls | 28 | 98 | 10.48 | 0.41 | 974.9 m | 154.9 m |
| OTK | Coordination | Boys | 32 | 168 | 10.34 | 0.35 | 2.22 m/s | 0.31 m/s |
| OTK | Coordination | Girls | 27 | 100 | 10.46 | 0.41 | 2.13 m/s | 0.29 m/s |
| OTK | Speed | Boys | 32 | 169 | 10.35 | 0.34 | 4.81 m/s | 0.43 m/s |
| OTK | Speed | Girls | 28 | 102 | 10.46 | 0.41 | 4.59 m/s | 0.44 m/s |
| OTK | PowerLOW | Boys | 32 | 169 | 10.35 | 0.35 | 139.8 cm | 20.5 cm |
| OTK | PowerLOW | Girls | 28 | 102 | 10.48 | 0.41 | 125.5 cm | 21.6 cm |
| OTK | PowerUP | Boys | 32 | 169 | 10.35 | 0.35 | 4.56 m | 0.75 m |
| OTK | PowerUP | Girls | 28 | 104 | 10.46 | 0.40 | 4.06 m | 0.73 m |
| OTK | Balance | Boys | 32 | 168 | 10.34 | 0.34 | 17.7 s | 15.4 s |
| OTK | Balance | Girls | 28 | 101 | 10.46 | 0.40 | 20.9 s | 17.2 s |

Keyage = Children with school enrollment according to key date, OTK = Older-than-keyage children (i.e., delayed school enrollment or repetition of a grade). Component = Physical fitness component. Endurance = cardiorespiratory endurance (i.e., 6-min run test), coordination = star-run test, speed = 20-m linear sprint test, powerLOW = lower limbs muscle power (i.e., standing long jump test), powerUP = upper limbs muscle power (i.e., ball push test), Balance = one-legged-stance test with eyes closed. Score (*M*) = Mean test score, Score (*SD*) = Standard deviation of test scores.

**Table S3** Sample description of keyage and OTK children in fifth grade.

| *Group* | *Component* | *Sex* | *N Schools* | *N Children* | *Age (M)* | *Age (SD)* | *Score (M)* | *Score (SD)* |
|---|---|---|---|---|---|---|---|---|
| Keyage | Endurance | Boys | 33 | 420 | 10.58 | 0.27 | 1077.6 m | 174.5 m |
| Keyage | Endurance | Girls | 33 | 453 | 10.56 | 0.29 | 1021.9 m | 129.8 m |
| Keyage | Coordination | Boys | 33 | 437 | 10.58 | 0.27 | 2.39 m/s | 0.29 m/s |
| Keyage | Coordination | Girls | 33 | 470 | 10.56 | 0.29 | 2.35 m/s | 0.27 m/s |
| Keyage | Speed | Boys | 33 | 444 | 10.58 | 0.27 | 4.97 m/s | 0.52 m/s |
| Keyage | Speed | Girls | 33 | 463 | 10.57 | 0.29 | 4.92 m/s | 0.43 m/s |
| Keyage | PowerLOW | Boys | 33 | 444 | 10.58 | 0.27 | 146.0 cm | 22.1 cm |
| Keyage | PowerLOW | Girls | 33 | 478 | 10.56 | 0.29 | 141.3 cm | 20.0 cm |
| Keyage | PowerUP | Boys | 33 | 441 | 10.58 | 0.27 | 4.95 m | 0.75 m |
| Keyage | PowerUP | Girls | 33 | 475 | 10.56 | 0.29 | 4.56 m | 0.68 m |
| Keyage | Balance | Boys | 33 | 432 | 10.57 | 0.27 | 19.9 s | 17.0 s |
| Keyage | Balance | Girls | 33 | 469 | 10.57 | 0.29 | 26.9 s | 19.9 s |
| OTK | Endurance | Boys | 33 | 136 | 11.42 | 0.35 | 1055.0 m | 171.9 m |
| OTK | Endurance | Girls | 28 | 75 | 11.45 | 0.38 | 973.8 m | 155.2 m |
| OTK | Coordination | Boys | 33 | 140 | 11.42 | 0.37 | 2.38 m/s | 0.33 m/s |
| OTK | Coordination | Girls | 28 | 79 | 11.48 | 0.39 | 2.28 m/s | 0.28 m/s |
| OTK | Speed | Boys | 33 | 142 | 11.41 | 0.34 | 5.02 m/s | 0.47 m/s |
| OTK | Speed | Girls | 28 | 79 | 11.47 | 0.38 | 4.93 m/s | 0.49 m/s |
| OTK | PowerLOW | Boys | 33 | 144 | 11.41 | 0.35 | 146.0 cm | 22.0 cm |
| OTK | PowerLOW | Girls | 27 | 81 | 11.48 | 0.39 | 138.4 cm | 22.7 cm |
| OTK | PowerUP | Boys | 33 | 143 | 11.40 | 0.34 | 5.10 m | 0.94 m |
| OTK | PowerUP | Girls | 28 | 81 | 11.48 | 0.39 | 4.62 m | 0.87 m |
| OTK | Balance | Boys | 33 | 137 | 11.42 | 0.38 | 20.5 s | 18.0 s |
| OTK | Balance | Girls | 28 | 77 | 11.48 | 0.38 | 25.5 s | 21.0 s |

Keyage = Children with school enrollment according to key date, OTK = Older-than-keyage children (i.e., delayed school enrollment or repetition of a grade). Component = Physical fitness component. Endurance = cardiorespiratory endurance (i.e., 6-min run test), coordination = star-run test, speed = 20-m linear sprint test, powerLOW = lower limbs muscle power (i.e., standing long jump test), powerUP = upper limbs muscle power (i.e., ball push test), Balance = one-legged-stance test with eyes closed. Score (*M*) = Mean test score, Score (*SD*) = Standard deviation of test scores.

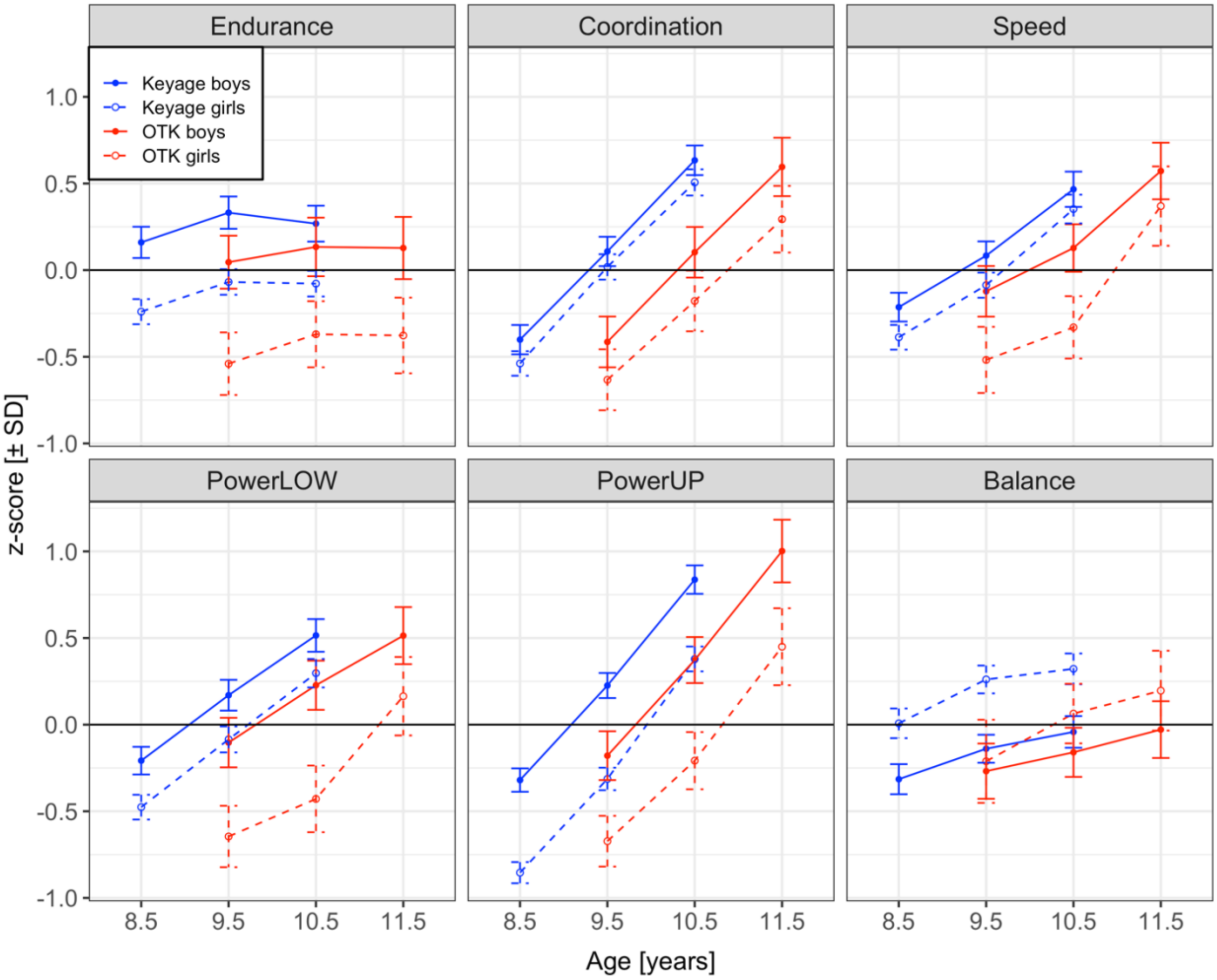


**Figure S1** Age and sex effects on physical fitness of keyage and OTK children. Performance (means and 95% CIs) in six physical fitness tests for keyage boys and girls (blue) and OTK boys and girls (red). The lines represent the physical fitness development from third to fourth, and from fourth to fifth grade. Keyage children were approximately 8.5 years in third, 9.5 years in fourth, and 10.5 years in fifth grade, OTK children were approximately 9.5 years in third, 10.5 years in fourth, and 11.5 years in fifth grade. Data were z-transformed. Endurance = cardiorespiratory endurance (i.e., 6-minute-run test), Coordination = star-run test, Speed = 20-m linear sprint test, PowerLOW = lower limbs muscle power (i.e., standing long jump test), PowerUP = upper limbs muscle power (i.e., ball-push test), Balance = static balance (i.e., one-legged-stance test with eyes closed). For coordination and speed, scores were converted from seconds to meters/seconds (i.e., pace scores; star-run test = 50.912 [m] / time [s]; 20-m linear sprint test = 20 [m] / time [s]). For static balance, scores were log-transformed. For all physical fitness tests, a larger z-score indicates a better physical fitness.
